# Comparison of left ventricle mechanical dyssynchrony parameters in ischemic and non-ischemic patients using _13_N-NH_3_ PET/CT

**DOI:** 10.1007/s12350-020-02466-w

**Published:** 2021-01-04

**Authors:** Angelica Mazzoletti, Domenico Albano, Francesco Bertagna, Claudio Tinoco Mesquita, Raffaele Giubbini

**Affiliations:** 1 Nuclear Medicine, University of Brescia and Spedali Civili Brescia, Brescia, Italy; 2 Universidade Federal Fluminense, Niterói, RJ Brazil

**Keywords:** Dyssynchrony, ammonia, PET/CT, 13N-NH3 PET/CT, regadenoson, myocardial perfusion, myocardial blood flow

## Abstract

**Background-Aim:**

The relationship between perfusion pattern and stress-induced changes in Left Ventricular Mechanical Dyssynchrony (LVMD) has been previously described with controversial results using stress-rest perfusion imaging studies. The aim of this study was to assess the relationship between perfusion pattern and stress-induced changes in LVMD usingo regadenoson/rest13N-NH3 PET/CT.

**Methods:**

There were 74 patients who underwent stress-rest 13N-NH PET/CT from January 2014 to October 2018 excluding patients with left bundle branch block, ventricular pacing and myocardial necrosis. The patients were divided into those with reversible perfusion defects at stress (Ischemic group, *n* = 18) and patients without reversible perfusion defects (non-ischemic group, *n* = 56). The LVMD parameters included: phase standard deviation (PSD) and phase histogram bandwidth (PHB), after stress and at rest. The ΔPSD (post-stressPSD-restPSD) and ΔPHB (post-stressPHB—restPHB) were calculated to measure stress-induced changes in LVMD.

**Results:**

There were no significant differences in LVMD parameters between post-stress and at rest in both groups. The PSD post-stress, ΔPSD and PHB post-stress were significantly higher in the ischemic group.

**Conclusions:**

Using a vasodilator as a stress, the PSD and PHB post-stress and ΔPSD were significantly higher in the ischemic patients than the non-ischemic group, while there were no significant differences in each cohort between stress and rest indices.

**Supplementary Information:**

The online version of this article (10.1007/s12350-020-02466-w) contains supplementary material, which is available to authorized users.

## Introduction

Left ventricular mechanical dyssynchrony (LVMD) is defined as the differences in the timing of onset of contraction between the different myocardial segments and it may have prognostic value for risk stratification.^1^,^2^ LVMD by phase analysis of gated myocardial perfusion imaging (MPI) has emerged as a robust, automated, and reproducible technique to quantify mechanical dispersion.^3^ 13N-ammonia (_13_N-NH_3_) may be used in PET/TC imaging and allows quantification of coronary flow reserve (CFR), myocardial blood flow (MBF) LV perfusion, wall motion, LV function and LVMD in patients with Coronary artery disease (CAD). Previous studies^4^–^6^ with different radiotracers such as Rubidium-82 and 99mTc-sestamibi, demonstrated that stress-induced ischemia causes dyssynchronous contraction in the ischemic region, leading to worsening of LVMD, but no previous study has examined the use of _13_N-NH_3_. The aim of this study was to evaluate the relationship between perfusion pattern and stress-induced changes in LVMD using _13_N-NH_3_ PET/CT after regadenoson stress and at rest.

## Methods

### PET/CT Imaging and Interpretation

Patients were in fasting state for stress studies. Maximal vasodilatation was obtained after I.V. injection of 400 mg of regadenoson over 10 seconds in the right antecubital vein followed by a bolus administration of a standard dose of 370 MBq of 13NH3 40 seconds after the end of regadenoson injection. The PET studies were acquired in 3D and list mode for 10 minutes starting acquisition immediately before 13NH3 injection by a discovery PET/CT 690 (GE Healthcare, Milwaukee, h Wisconsin, USA). A low-dose CT attenuation correction (140 kV, 120-150 mA) was acquired for optimal imaging position on a CT scout scan and for attenuation correction. PET images were corrected for attenuation. The reconstruction was performed using iterative algorithms OSEM (Ordered-subset expectation maximization) with 3 iterations and 24 subsets, filter cut-off 6 mm and 128 × 128-pixel matrix). Gated images were reconstructed in 16 bins and the dynamic images and in 32 frames for both stress and rest (24 × 10s, 4 × 30s, 4 × 60s). The perfusion pattern was assessed after iterative reconstruction of tomographic slices and evaluated as recommended by the American Society of Nuclear Cardiology. The SDS was determined by 4-DM Corridor software package (INVIA, Ann Arbor, Michigan), using an Institutional gender-matched normal database. We calculated the SSS and the SRS as the sum of the respective scores of all 17 segments, and derived SDS as the difference between SSS and SRS, served as a measure of reversibility. A SDS ≤ 1 was considered as normal.^7^ Aminophylline was injected at the end of the stress part (240 mg in 10 mL). Stress examination was performed with continuous ECG monitoring to assess heart rate increase induced by regadenoson stimulation. Two hours after the stress examination, rest studies were acquired for 10 minutes in 3D and list mode after the injection of a standard dose of 370 MBq of _13_N-NH_3_. Quantitative MBF and CFR were determined using the PMOD software package (PMOD Technologies Ltd., Zurich, Switzerland). The CFR was calculated as the ratio of hyperemic to resting MBF; CFR e” 2.5 was considered as normal. The SDS as well as the MBF and CRF was evaluated for the global left ventricle (LV) and for the three coronary territories using a 17-segment model according to the American Society of Nuclear Cardiology recommendations. The PMode software package provides automatically measures of rest, stress MBF, and CFR for global, segmental (according to the 17 segment model), and for the three main vascular territories, identified as anterior, anteroseptal, and apical segments for the left anterior descending coronary artery, inferior and infero-septal segments for the right coronary artery, and of lateral segments for the left circumflex, respectively.

### Patient Features

Seventy-four patients (44 men; 30 women), average age 62 ± 12.,72 (range 36-89) who had stress-rest _13_N-NH_3_ PET/CT from January 2014 to October 2018 at our department were enrolled. Patients with left bundle branch block, ventricular pacing, previous revascularization and myocardial necrosis were excluded from the study. Patients were divided into two groups accordingly MPI results: those with reversible myocardial perfusion defects at stress (ischemic group, n. 18) and those without reversible perfusion defects (non ischemic group, n. 56). In each group, we compared the demographics such as age, sex, smoke history, obesity (evaluated as BMI >30), history of hypertension, diabetes, hyperlipidemia and kidney failure. LVMD parameters included phase histogram bandwidth (PHB) and phase standard deviation (PSD). PHB represents the range of degrees of the cardiac cycle during which 95% of myocardium is starting to contract; PSD is the standard deviation of the range. The ΔPSD (evaluated as the difference between post- stress PSD and rest PSD) and ΔPHB (- the difference between post- stress PHB and PHB at rest) were calculated to measure stress-induced changes in LVMD.

### Statistical Analysis

Statistical analyses were performed out using MedCalc Software version 18.1. The descriptive analysis of categorical variables are characterized by the calculation of simple and relative frequencies, while the numeric variables by median, mean, minimum and maximum values.

Either student’s t test or Mann–Whitney test was used to compare quantitative data between different groups, when required. Chi square (*χ*2) test was used to compare proportions. A *P* value < 0.05 was considered to indicate statistical significance.

## Results

**Figure 1:**
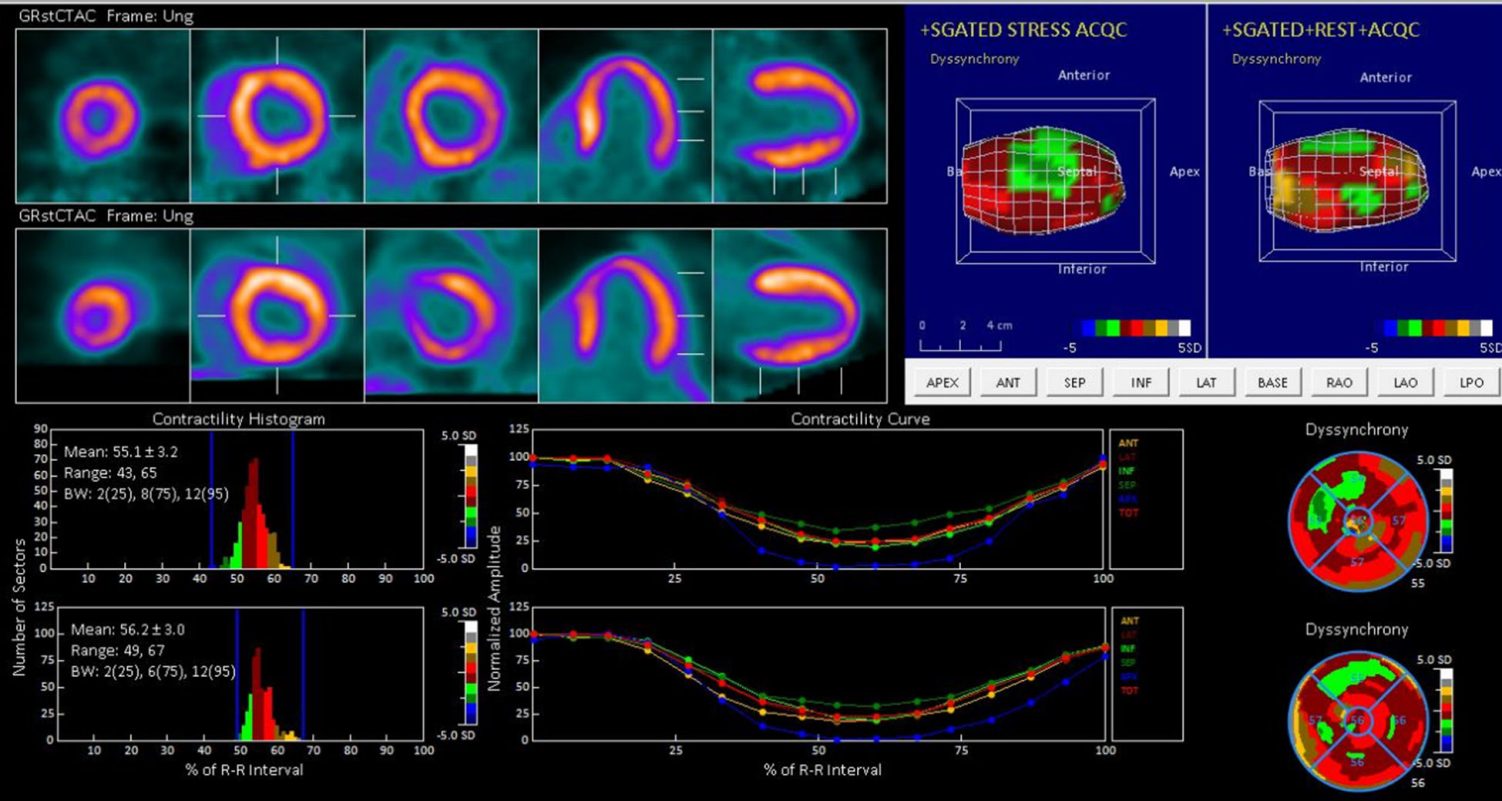
A representative case of Ischemic Patient. Woman, 75- years old, without history of cardiovascular risk factors

**Figure 2:**
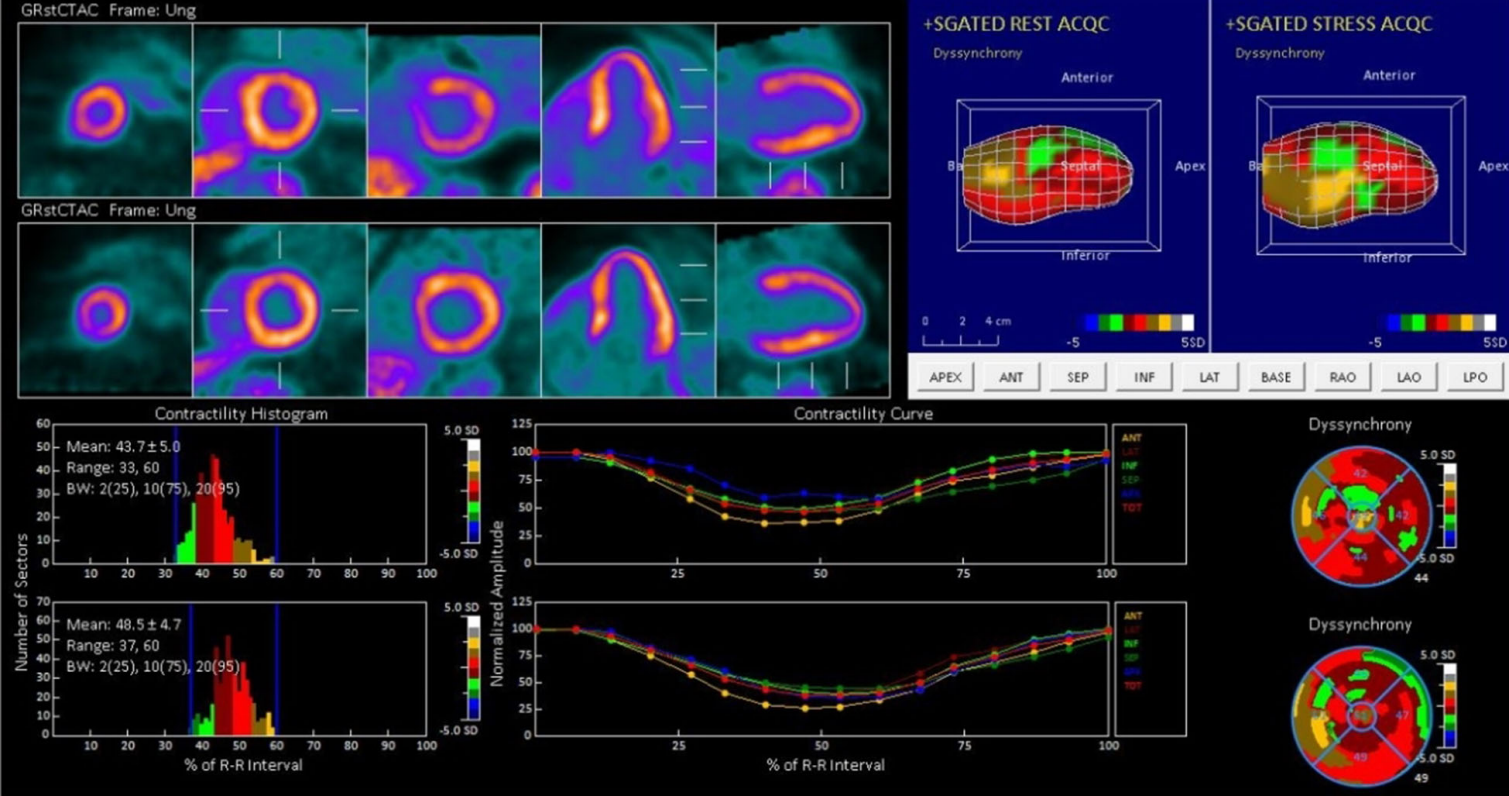
A representative case of Non- Ischemic Patient. Man, 52 years old, without history of cardiovascular risk factors

The demographics are in Table 1. Non significant differences were observed between ischemic and non-ischemic groups except of sex and history of diabetes. The LVMD parameters are in Table 2. The LVMD parameters (PSD and PHB) were not significant differences between stress and rest in each group. All LVMD parameters were higher in the ischemic patients compared to non- ischemic ones, but only PSD post- stress—ΔPSD—and PHB post stress were statistically significant. The correlation matrix between LVMD and perfusion pattern is shown in Table 3. Examples from both groups are shown in Figures 1 and 2.

**Table 1:** Epidemiological and clinical parameters between ischemic and non-ischemic patients

	Ischemic group (18)	Non ischemic group (56)	*P* Value
Average age	67±18	63 ± 13	
Men	15/18	29/56	0.017
Women	3/18	27/56	
Hypertension	9/18	34/56	0.29
Hyperlipidemia	9/18	28/56	0.5
Diabetes	6/18	9/56	0.04
BMI>30	3/18	7/56	0.39
Kidney failure	1/18	2/56	
Smoke	3/18	14/56	0.68
Smoke in the past	5/18	16/56	0.45

**Table 2:** Left ventricle mechanical dyssynchrony parameters between ischemic and non-ischemic patients

	Ischemic group (18)	Non ischemic group (56)	*P* Value
PSD stress	6.01±6.57	4.16±2.68	0.003
PSD rest	4.6±2.8	4.28±1.06	0.48
ΔPSD	1.41±3.74	− 0.11±1.62	0.003
PHB stress	24.22±32.52	17.46±7.07	0.008
PHB rest	18.55±14.14	15.67±5.65	0.09
ΔPHB	5.66±18.38	1.78±1.41	0.07
MBF stress	1.9±0.03	2.4±3.4	0.04

**Table 3:** Correlation between dyssynchrony parameters with perfusion parameters in different groups

	Entire population *n* 74		Ischemic group *n* 18		Non-ischemic group *n* 56	
	Correlation coefficient	*P* Value	Correlation coefficient	*P* Value	Correlation coefficient	*P* Value
PSD stress vs SSS	0.264	0.059	− 0.001	0.998	0.294	0.098
PHB stress vs SSS	0.227	0.064	− 0.036	0.890	0.276	0.056
ΔPSD vs SDS	0.134	0.277	0.021	0.934	− 0.174	0.224
ΔPHB vs SDS	0.178	0.148	0.080	0.759	0.064	0.657
ΔPSD vs CFR	− 0.134	0.252	− 0.372	0.128	0.083	0.536
ΔPHB vs CFR	− 0.120	0.306	− 0.221	0.377	− 0.012	0.928

## Discussion

Intraventricular dyssynchrony reflects inhomogeneous timing of contraction of different myocardial segments, caused by disturbed myocyte stimulation or impaired contractility.^8^,^9^ It is helpful to recognize that even structurally normal hearts exhibit some degree of non-uniformity in contraction due to its complex spatial and geometric architecture. Contraction movements depend on the complex distribution of myocardial fibers within the epicardial and endocardial regions as they are oriented longitudinally through the long axis of the heart and circumferentially within the mid-wall region. This arrangement allows for a complex contractile movement which involves both longitudinal and circumferential fibers from apex to base during systolic activation. Due to this complex fiber architecture and to the presence of His-Purkinje system, which allow electrical activation, systolic contraction can be well executed allowing efficient pump function. It is not surprising that in an ischemic heart abnormal temporal electrical activation of the complex myocardial fiber architecture reduces pump efficiency and cardiac performance.^8^ As a result of that abnormal activation loading, LVMD parameters increase and reflects a balance of forces, with the region that is activated early being unable to withstand the stress generated by the late-activated LV segments.^10^–^12^ The regional wall contractions are not effectively converted to pressure build-up in the left ventricle, but rather cause substantial blood volume shifts within the LV cavity. The overall result is a decrease in LV pumping efficiency.^9^–^12^ Several new imaging techniques are proving useful for diagnosis of LV dyssynchrony and PET/TC represents a useful method to quantify LVMD parameters. Different radiotracers allow the study of both myocardial perfusion and myocardial metabolism such as radioactive _13_N-NH_3_, H_2_O_15_, _82_Rb. While water is freely diffusible without being retained from myocardial tissue, ammonia and rubidium present different pharmacokinetic features and they are usually retained in the myocardial tissue depending on myocardial blood flow. While rubidium crosses the myocyte cell membrane mainly by active diffusion, ammonia crosses the myocyte cell membrane by passive diffusion. Unlike technetium radiotracers, ammonia-PET/CT images are acquired immediately after stress induction by regadenoson injection, at maximum peak of vascular dilatation and it allow for the evaluation of absolute myocardial perfusion and mechanical synchrony at real peak hyperemic stress. Myocardial extraction of the PET/TC radiotracers at rest is higher than the radiotracers used with SPECT and it depends on myocardial blood flow. When compared to adenosine, regadenoson has been shown to be non-inferior for identifying perfusion defects^13^ and providing prognostic data.^14^–^19^ Our study shows that LVMD is different in ischemic and non-ischemic patients though in each group they were comparable at rest and post stress. The study has limitations as it is retrospective and the ischemic group has small number of patients. However, it is a proof of principle that our protocol could be used to study LVMD in addition to other variables such as perfusion, function and MBF.

## New Knowledge Gained

13N-NH3 PET/TC represent a reliable method for estimating myocardial dyssynchrony parameters. The presence of ischemia is confirmed by a non-synchronous contraction of myocardial tissue and a decrease of myocardial blood flow, parameters evaluated after pharmacological stress test conducted with the use of regadenoson.

## Electronic supplementary material

Below is the link to the electronic supplementary material.

(PPT 3525 kb)
