# Supplementary material for: Comparison of left ventricle mechanical dyssynchrony parameters in ischemic and non-ischemic patients using 13N-NH3 PET/CT
Source: J Nucl Cardiol. 2021 Jan 4;29(3):1248–53. doi: 10.1007/s12350-020-02466-w (PMC9163010; doi:10.1007/s12350-020-02466-w)
Supplement: Supplementary file 1 — (PPT 3525 kb) [file 12350_2020_2466_MOESM1_ESM.ppt]

## Slide 1
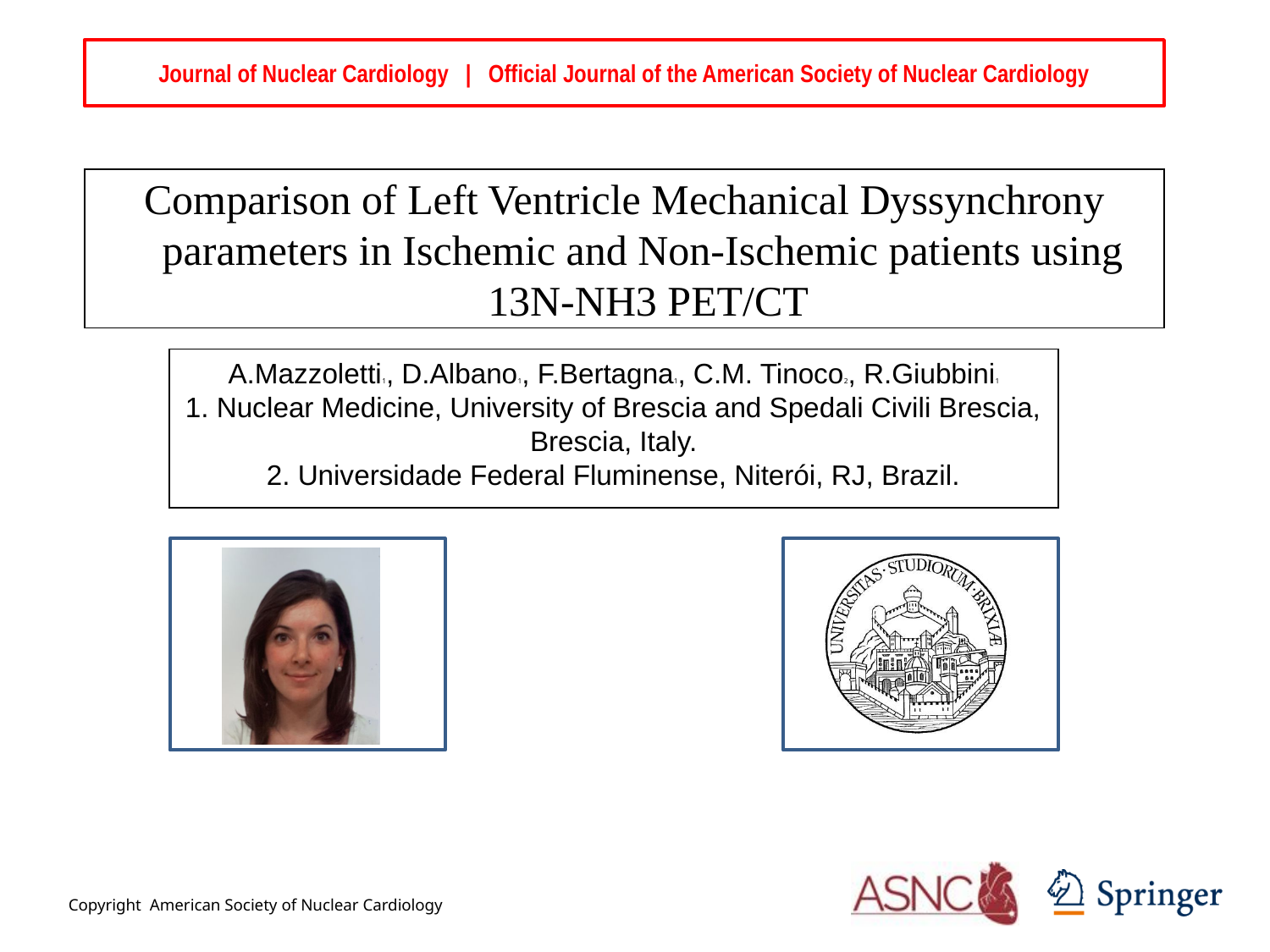

Journal of Nuclear Cardiology | Official Journal of the American Society of Nuclear Cardiology
Comparison of Left Ventricle Mechanical Dyssynchrony parameters in Ischemic and Non-Ischemic patients using 13N-NH3 PET/CT
A.Mazzoletti1, D.Albano1, F.Bertagna1, C.M. Tinoco2, R.Giubbini1
1. Nuclear Medicine, University of Brescia and Spedali Civili Brescia, Brescia, Italy.
2. Universidade Federal Fluminense, Niterói, RJ, Brazil.
Copyright American Society of Nuclear Cardiology

## Slide 2
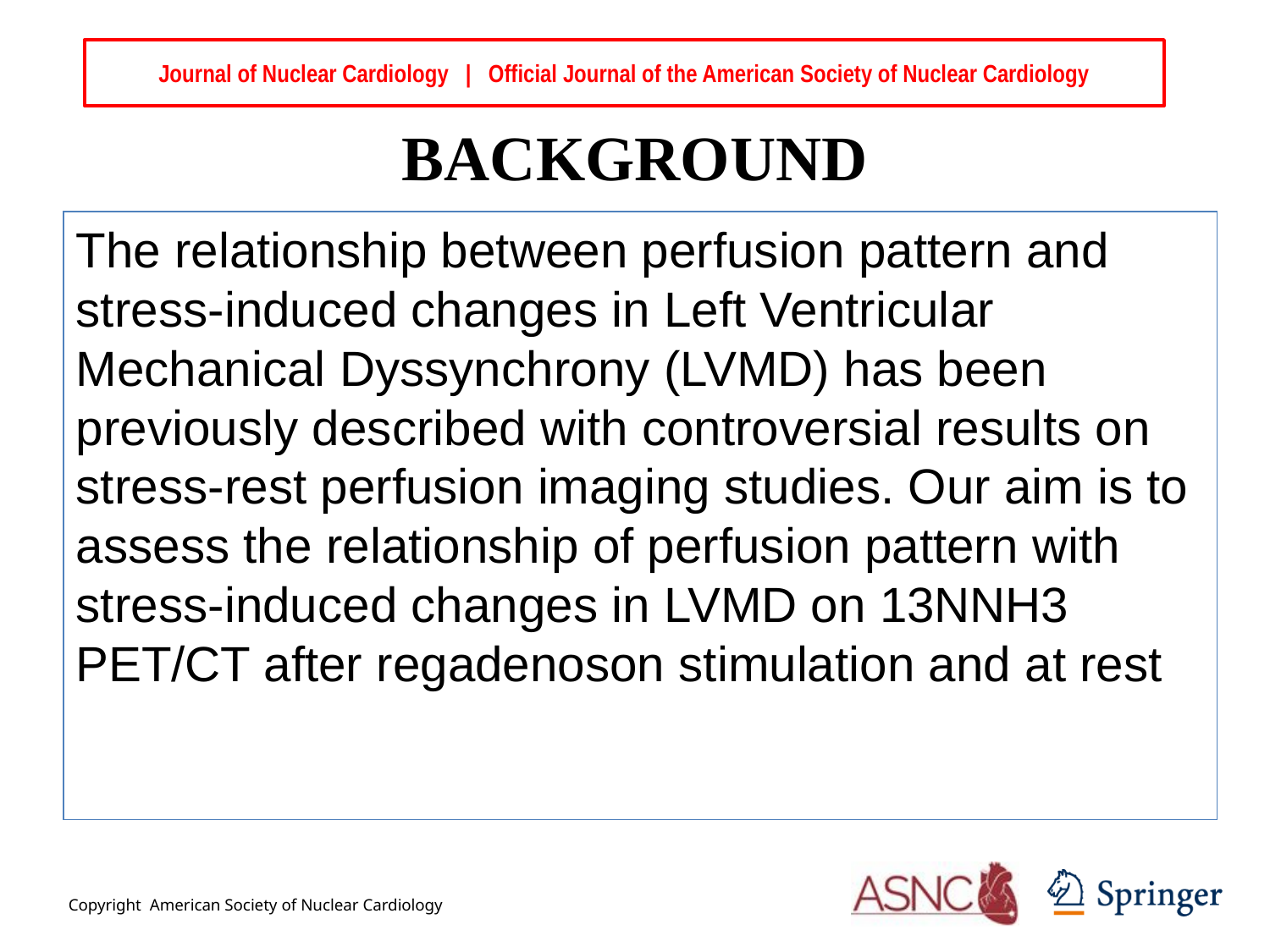

Journal of Nuclear Cardiology | Official Journal of the American Society of Nuclear Cardiology
BACKGROUND
The relationship between perfusion pattern and stress-induced changes in Left Ventricular Mechanical Dyssynchrony (LVMD) has been previously described with controversial results on stress-rest perfusion imaging studies. Our aim is to assess the relationship of perfusion pattern with stress-induced changes in LVMD on 13NNH3 PET/CT after regadenoson stimulation and at rest
Copyright American Society of Nuclear Cardiology

## Slide 3
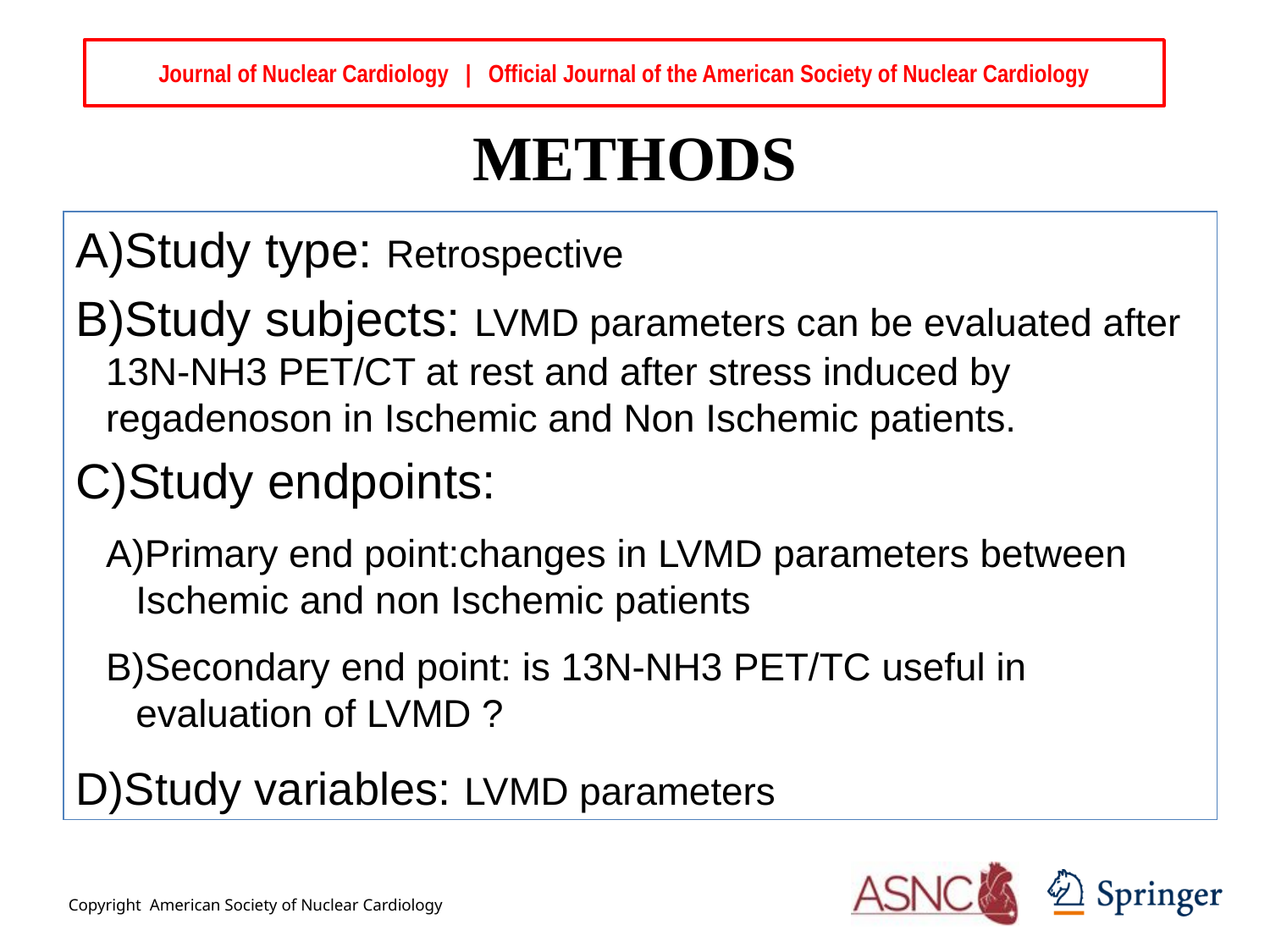

Journal of Nuclear Cardiology | Official Journal of the American Society of Nuclear Cardiology
METHODS
Study type: Retrospective
Study subjects: LVMD parameters can be evaluated after 13N-NH3 PET/CT at rest and after stress induced by regadenoson in Ischemic and Non Ischemic patients.
Study endpoints:
Primary end point:changes in LVMD parameters between Ischemic and non Ischemic patients
Secondary end point: is 13N-NH3 PET/TC useful in evaluation of LVMD ?
Study variables: LVMD parameters
Copyright American Society of Nuclear Cardiology

## Slide 4
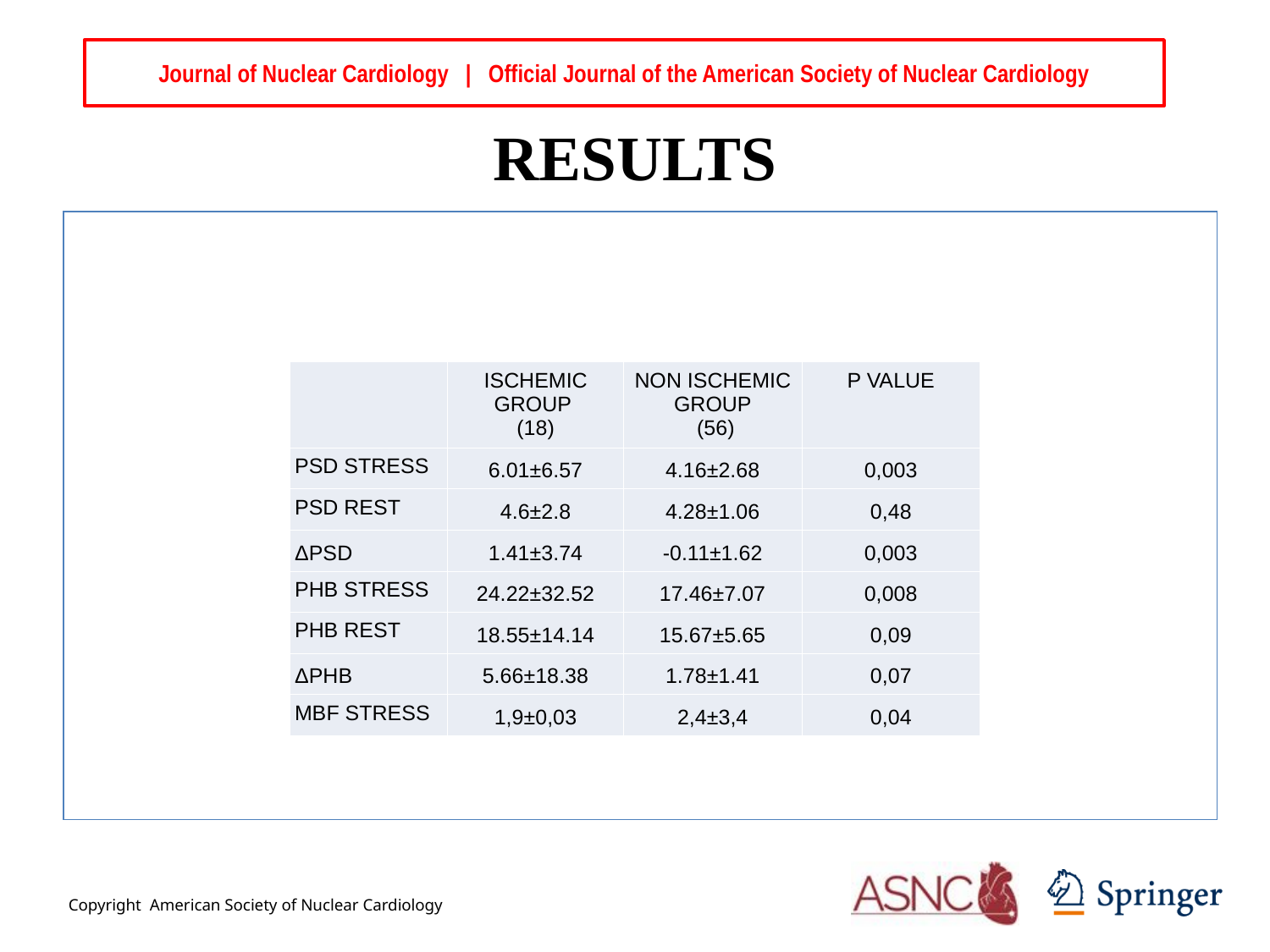

Journal of Nuclear Cardiology | Official Journal of the American Society of Nuclear Cardiology
RESULTS
| | ISCHEMIC GROUP (18) | NON ISCHEMIC GROUP (56) | P VALUE |
| --- | --- | --- | --- |
| PSD STRESS | 6.01±6.57 | 4.16±2.68 | 0,003 |
| PSD REST | 4.6±2.8 | 4.28±1.06 | 0,48 |
| ΔPSD | 1.41±3.74 | -0.11±1.62 | 0,003 |
| PHB STRESS | 24.22±32.52 | 17.46±7.07 | 0,008 |
| PHB REST | 18.55±14.14 | 15.67±5.65 | 0,09 |
| ΔPHB | 5.66±18.38 | 1.78±1.41 | 0,07 |
| MBF STRESS | 1,9±0,03 | 2,4±3,4 | 0,04 |
Copyright American Society of Nuclear Cardiology

## Slide 5
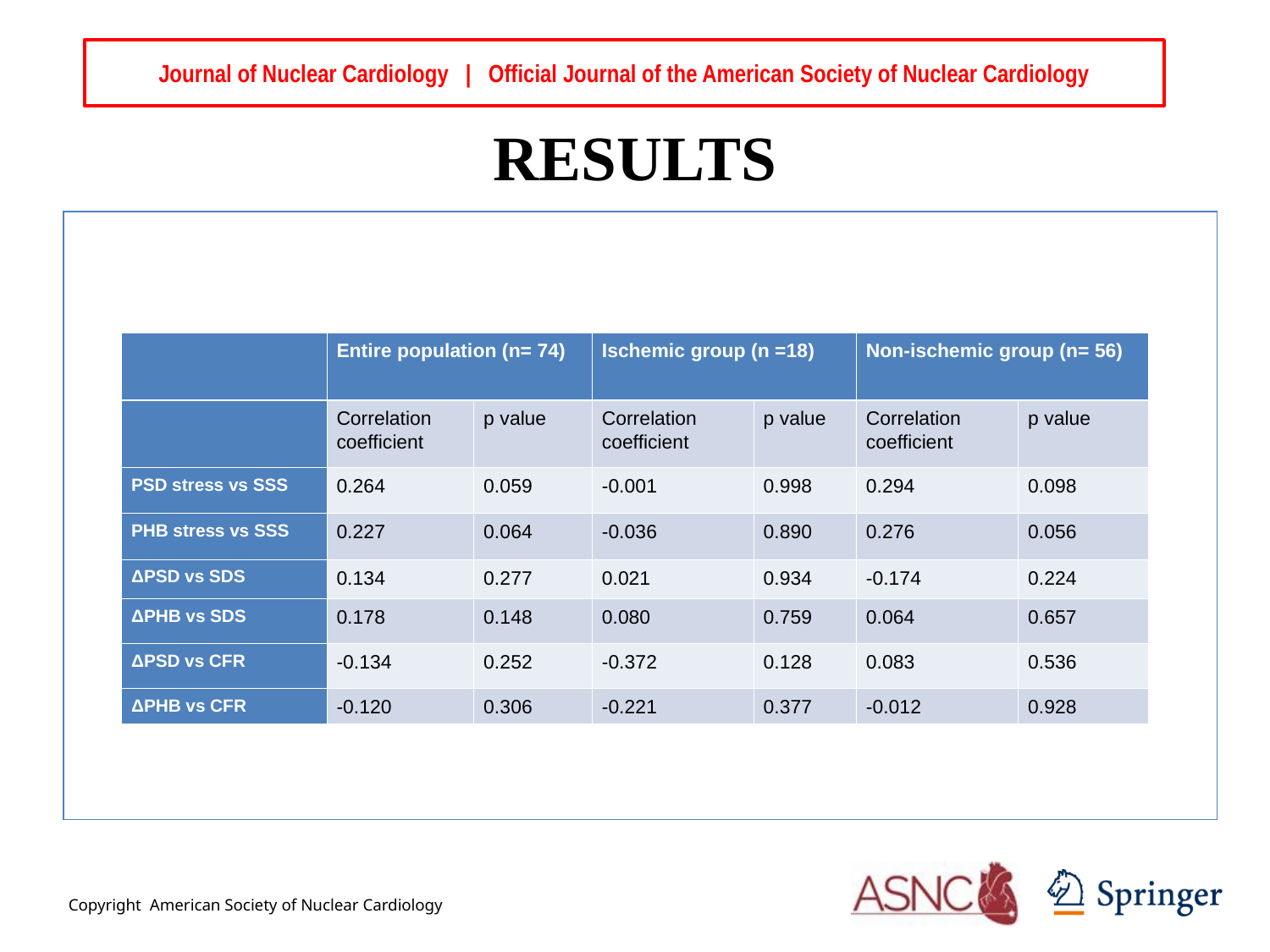

Journal of Nuclear Cardiology | Official Journal of the American Society of Nuclear Cardiology
RESULTS
| | Entire population (n= 74) | | Ischemic group (n =18) | | Non-ischemic group (n= 56) | |
| --- | --- | --- | --- | --- | --- | --- |
| | Correlation coefficient | p value | Correlation coefficient | p value | Correlation coefficient | p value |
| PSD stress vs SSS | 0.264 | 0.059 | -0.001 | 0.998 | 0.294 | 0.098 |
| PHB stress vs SSS | 0.227 | 0.064 | -0.036 | 0.890 | 0.276 | 0.056 |
| ΔPSD vs SDS | 0.134 | 0.277 | 0.021 | 0.934 | -0.174 | 0.224 |
| ΔPHB vs SDS | 0.178 | 0.148 | 0.080 | 0.759 | 0.064 | 0.657 |
| ΔPSD vs CFR | -0.134 | 0.252 | -0.372 | 0.128 | 0.083 | 0.536 |
| ΔPHB vs CFR | -0.120 | 0.306 | -0.221 | 0.377 | -0.012 | 0.928 |
Copyright American Society of Nuclear Cardiology

## Slide 6
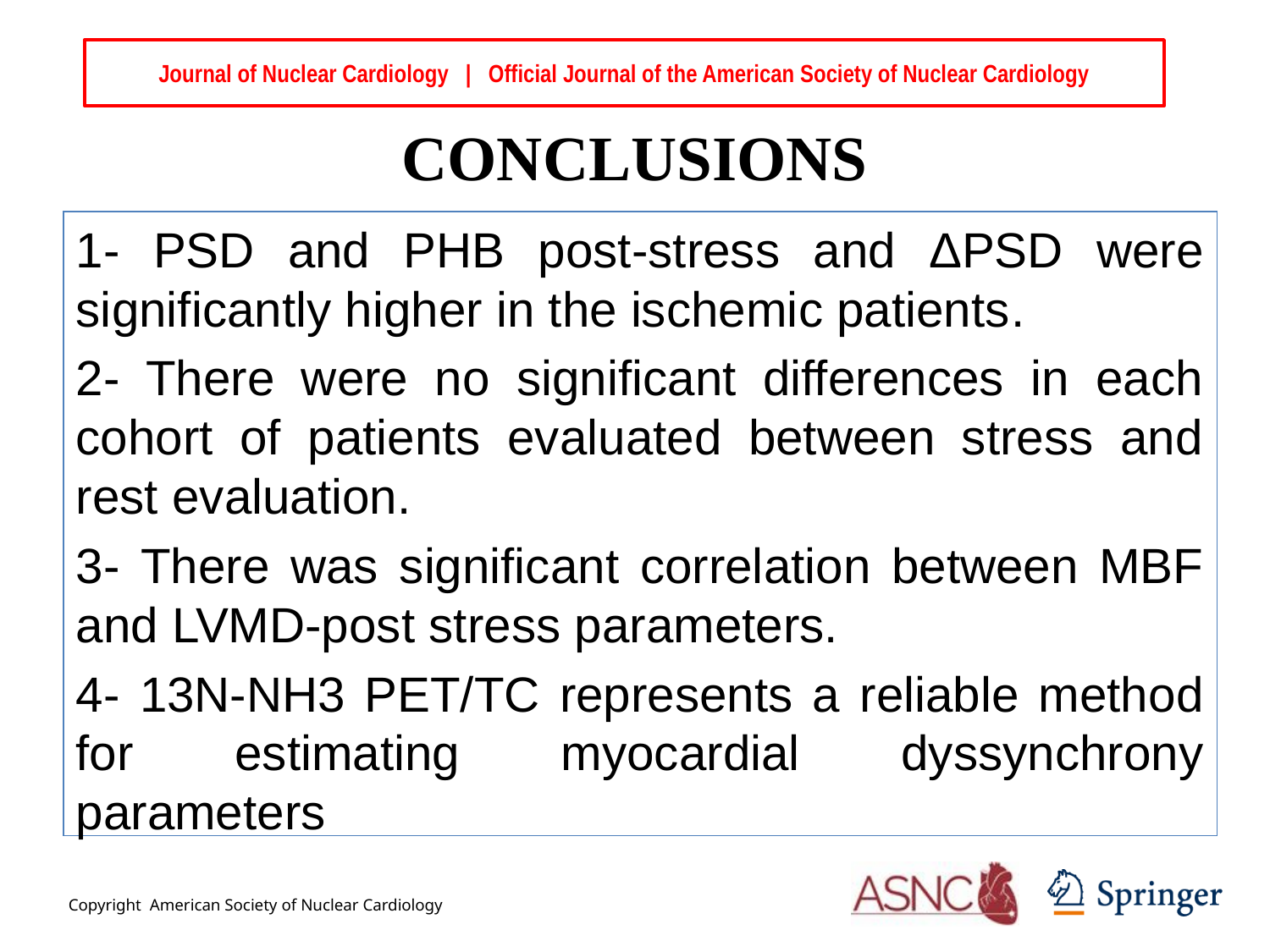

Journal of Nuclear Cardiology | Official Journal of the American Society of Nuclear Cardiology
CONCLUSIONS
1- PSD and PHB post-stress and ΔPSD were significantly higher in the ischemic patients.
2- There were no significant differences in each cohort of patients evaluated between stress and rest evaluation.
3- There was significant correlation between MBF and LVMD-post stress parameters.
4- 13N-NH3 PET/TC represents a reliable method for estimating myocardial dyssynchrony parameters
Copyright American Society of Nuclear Cardiology
